# The impact of the recent HIV non-nucleoside reverse transcriptase inhibitors and nucleoside reverse transcriptase inhibitors-based regimens on metabolic health outcomes: A narrative review

**DOI:** 10.1016/j.bbrep.2025.102345

**Published:** 2025-11-06

**Authors:** Amogelang Sedibe, Ntethelelo Sibiya, Trevor Nyakudya, Mlindeli Gamede

**Affiliations:** a Department of Physiology, Faculty of Health Sciences, University of Pretoria, Gauteng, South Africa; b Pharmacology Division, Faculty of Pharmacy, Rhodes University, Makhanda, Eastern Cape, South Africa

**Keywords:** HIV- antiretroviral therapy, HIV-Fixed-dose combination therapy, Prediabetes, Type 2 diabetes mellitus, Drug-metabolic complications, Pancreatic health outcomes

## Abstract

The global prevalence of human immunodeficiency virus (HIV) has led to a significant rise in the chronic use of antiretroviral (ARV) drugs, both for HIV management and pre-exposure prophylaxis (PrEP), to meet the set Joint United Nations Programme on HIV/AIDS (UNAIDS) 95-95-95 targets. Although antiretroviral therapy (ART) has remarkably increased the life expectancy of people living with HIV (PLHIV), it has also been associated with metabolic complications, particularly in glucose and lipid metabolism. Notably, the development of type two diabetes mellitus (T2DM), accounting for 90–95 % of diabetes cases, often stems from an asymptomatic prediabetic state, frequently left undiagnosed. In this narrative review, we address the limited understanding of how prediabetic individuals respond to chronic exposure to antiretroviral therapy. The scope of this review focuses on selected markers of pancreatic metabolic dysfunction, the interplay between modern ARV therapies and prediabetes will be examined. In efforts to enhance and further expand the understanding of potential risks and outcomes of ARVs on metabolically compromised individuals. Through a comprehensive synthesis of existing literature and novel findings from the animal model, *in vitro* studies and clinical studies, we aim to provide valuable insights for both the scientific and clinical communities, contributing to the optimization of HIV treatment strategies and the mitigation of associated metabolic complications. Based on the available literature, it is evident that more research is needed to better understand the interaction between prediabetes and ART in HIV-infected individuals, to simultaneously reach the set UNAIDS 95-95-95 HIV/AIDS targets and combat the rising trend of noncommunicable diseases in HIV-infected populations.

## Highlights

• The HIV-ART is one of the most effective medications in HIV infection management.
• The role of the current HIV-ART on carbohydrate metabolism.
• HIV-ART has been previously associated with changed metabolic complications.
• The prediabetes as a significant factor in the interplay between HIV and diabetes.
• The impact of HIV-ART on pancreatic function and future research on HIV-ART and pancreatic β-cells function.

## Graphical abstract

**Figure:**
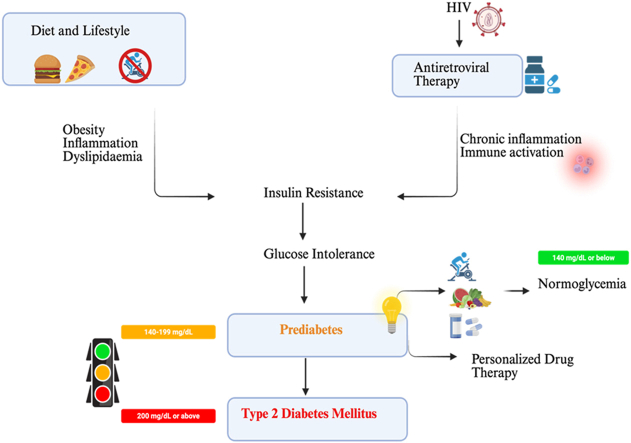

## 1 Review scope

This narrative review article examines the remarkable, however deleterious, effects of antiretroviral therapy (ART) that are currently used in HIV management and prevention. The information from all type of studies that report on the metabolic effects of NRTIs and NNRTIs will be considered for this review. ART has changed the trajectory of HIV infection through effective therapy; however, the associated metabolic complications are of high concern. This article identifies prediabetes as a significant factor in the interplay between HIV and diabetes. Furthermore, this article aims to prompt future research to investigate the effects of modern combination therapy in the pancreatic islets, particularly β-cells.

## 2 Introduction

The global epidemic of metabolic disorders increasingly intersects with Human Immunodeficiency Virus (HIV) infection. Although the introduction of highly active antiretroviral therapy (HAART) has remarkably increased the life expectancy of people living with HIV (PLHIV), both HIV infection and antiretroviral therapy (ART) have been independently and synergistically associated with a range of metabolic complications, particularly those involving glucose and lipid metabolism [[15], [16], [17]]. Over the past four decades since the initial case of HIV was discovered, the infection continues to pose a substantial public health challenge, with the Joint United Nations Programme on HIV/AIDS (UNAIDS) reporting approximately 39.9 million PLHIV in 2023 [18]. The burden of disease is particularly concentrated in low and middle income countries, with sub-Saharan Africa accounting for approximately two-thirds of the global HIV cases [19]. South Africa alone has an estimated HIV prevalence of approximately 12.7 % of its population, which translates to about 7.8 million PLHIV, making it the country with the highest prevalence globally [20]. In the fight against HIV epidemic, UNAIDS has set a global goal to end HIV/AIDS as a public health issue by 2030 [21]. To attain this goal, targets have been set for every step in HIV prevention and management strategies. Initially established as the three 90's, the strategy was updated in 2020 to the UNAIDS 95-95-95 targets, aimed at achieving approximately 95 % of the general population aware of their HIV status, 95 % of those diagnosed to receive ART, and 95 % of those on ART to achieve viral suppression [22]. In parallel with these efforts, HIV prevention strategies such as pre-exposure prophylaxis (PrEP) have expanded access to ART, leading to a growing number of individuals using antiretroviral drugs for both treatment and prevention [23,24]. Previous regimens and formulations of antiretroviral drugs introduced in the late 1990s have been associated with metabolic derangements including hyperlipidaemia, fat redistribution and type 2 diabetes mellitus (T2DM) [17]. The newly formulated fixed-dose combination ART regimens, typically administered as a single daily pill, have improved patient adherence, safety, and efficacy [25,26]. However, the metabolic derangements associated with the chronic treatment using these formulations remain unclear. This may be attributed to the lack of longitudinal studies investigating the long-term metabolic effects of these drugs [27]. The pathophysiological mechanisms underlying these ART-associated metabolic disorders remain relatively unclear and multifactorial; however, insulin resistance appears to play a central role by disrupting interconnected pathways involved in glucose and lipid metabolism [28]. This review identifies prediabetes as a significant factor in the interplay between HIV and diabetes. The metabolic burden of ART use is particularly concerning in the context of a global rise in lifestyle-linked conditions such as obesity and prediabetes. Prediabetes, an asymptomatic yet high-risk metabolic state, frequently goes undiagnosed and is associated with early pancreatic dysfunction, insulin resistance and impaired incretin responses [29]. Chronic ART usage may overlap with metabolic vulnerabilities in the ageing population [30]. Furthermore, this necessitates investigating the interaction between ART regimens and progressive loss of metabolic health, especially in individuals with pre-existing metabolic conditions such as prediabetes. This review hypothesises that modern fixed-dose combination ART regimens, particularly those composed of nucleoside reverse transcriptase inhibitors (NRTIs) and non-nucleoside reverse transcriptase inhibitors (NNRTIs), may contribute to early and progressive metabolic disturbances through mechanisms that affect pancreatic function and incretin hormone dynamics. These ART-related metabolic effects may be compounded in individuals already at risk due to obesity or prediabetes, conditions that are themselves rising in prevalence globally. This review aims to synthesize available evidence, highlight the relatively underexplored mechanistic links between ART, pancreatic pathology, and early markers of metabolic disease, and identify critical knowledge gaps. In doing so, it seeks to inform clinical decision-making for healthcare providers managing the aging HIV-affected population, for whom balancing viral suppression and long-term metabolic health is increasingly important for their overall well-being and quality of life.

## 3 Intersecting burdens: lifestyle-linked metabolic disease and ART exposure

### 3.1 Obesity as a precursor of metabolic diseases

Obesity is a well-established contributor to metabolic disorders, including insulin resistance and T2DM [31,32]. This condition is characterised by an imbalance between energy intake and energy expenditure, resulting in excessive fat accumulation that disrupts nearly all normal physiological functions of the body [33]. Obesity thereby poses a great risk to health and the overall quality of life for those affected, including both children and adults [34]. Traditionally, body mass index (BMI ≥30 kg/m^2^) has been used as the primary measure to define obesity [35]. However, other measures of adiposity such as waist circumference and body fat percentage have been recommended in combination with BMI to better assess associated risk factors and capture the multifactorial nature of obesity [32]. The global prevalence of obesity has reached epidemic proportions. According to the World Health Organization (WHO), approximately 890 million adults and 160 million children were estimated to be obese as of 2022 [36]. Moreover, these numbers are expected to exceed one billion individuals, more than half of the global population, by 2030 [37]. The pathogenesis of obesity is complex and multifactorial, involving synergistic interactions between genetic, epigenetic, and behavioural factors [38]. However, lifestyle-related risk factors, particularly high-calorie diets and physical inactivity, have been established as the primary drivers of obesity [32,38]. Chronic excess energy intake leads to an accumulation of fat that exceeds the energy-storage capacity of adipocytes, causing adipocyte hypertrophy and subsequent adipose tissue dysfunction [32]. The impaired ability of hypertrophic adipocytes to store excess energy is associated with the release of free fatty acids into circulation, promoting ectopic fat deposition in non-adipose organs such as the liver, pancreas and skeletal muscle [39]. Ectopic fat accumulation induces lipotoxicity, which disrupts organ function and contributes to the onset of several metabolic conditions such as T2DM and metabolic syndrome, all of which increase cardiovascular disease risk [33]. Accumulation of lipids in the liver and skeletal muscle induces insulin resistance, which leads to increased hepatic glucose production and reduced glucose uptake in skeletal muscle, thereby overstimulating the pancreatic β-cells [32].

### 3.2 ART and the intersecting burden of lifestyle-driven metabolic dysfunction

**Fig. 1:**
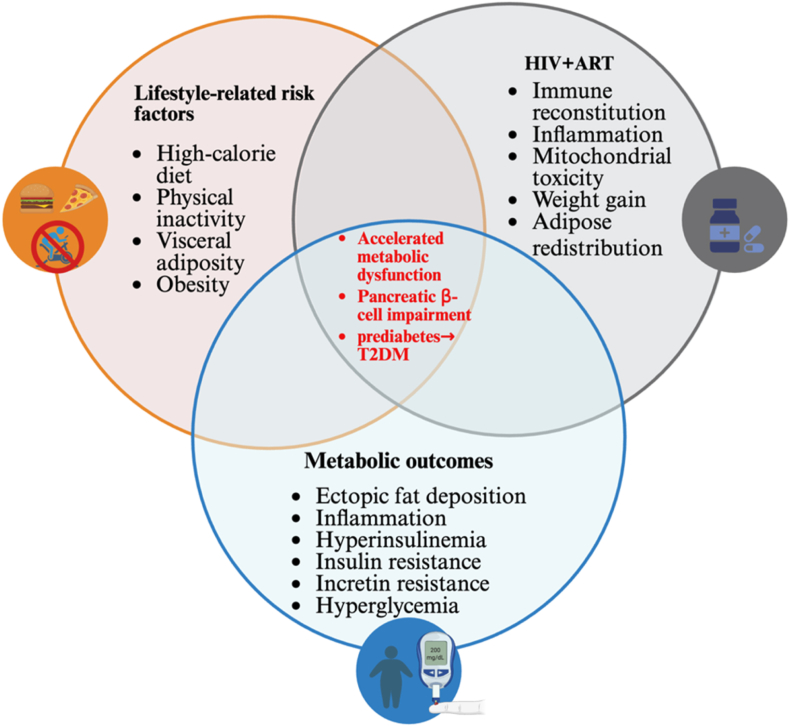
*ART and the intersecting burden of lifestyle-driven metabolic dysfunction (created in BioRender)*.

The growing burden of ART-related metabolic complications intersects with lifestyle-driven metabolic dysfunction, posing an amplified risk to the ageing HIV population, who are increasingly vulnerable to obesity, adipose dysfunction, and insulin resistance (Fig. 1) [40]. The introduction of effective ART has transformed HIV infection into a manageable chronic condition. However, emerging evidence suggests that ART has contributed to rising obesity rates among HIV-infected individuals [41]. The global prevalence of obesity among HIV-infected individuals remains underreported and varies widely by region due to geographical differences and heterogeneity in the population, which are closely linked to socioeconomic status, ethnicity, healthcare access, and behavioural factors [42,43]. Despite this variability, studies have shown that HIV-related obesity has become an increasingly significant public health challenge, with rates approaching parity or even higher than those of the general population [42]. HIV has been shown to directly impact adipose tissue by establishing reservoirs within immune cells such as CD4 T cells and macrophages [44,45]. These infected immune cells release viral proteins such as Nef and Tat, which contribute to adipose tissue dysfunction by impairing adipogenesis, promoting fibrosis through increased extracellular matrix production, and inducing local inflammation [46,47]. The initiation of effective ART may interact with these HIV-induced alterations, potentially exacerbating metabolic dysfunction [41,46]. Moreover, ART regimens containing tenofovir alafenamide (TAF) are linked to more pronounced increases in weight compared to those containing tenofovir disoproxil fumarate (TDF) [5,48]. Among the non-nucleoside reverse transcriptase inhibitors (NNRTIs), rilpivirine has been associated with greater weight gain than efavirenz [49]. Long-term ART exposure is also associated with progressive adiposity, exacerbating anthropometric changes accompanied by ART initiation and increasing the risk of HIV patients transitioning from normal (18.5–24.9 kg/m^2^) or overweight BMI (25.0–29.9 kg/m^2^) to obesity (BMI ≥30 kg/m^2^) [50]. This excess adiposity, characterised by adipocyte hypertrophy, results in the dysregulated secretion of adipokines and the release of free fatty acids, which impair insulin signalling and contribute to metabolic complications [51,52]. Moreover, hypertrophic adipocytes undergo injury and cellular stress, triggering the recruitment of macrophages to the adipose tissue microenvironment [53]. This immune infiltration promotes the release of pro-inflammatory cytokines such as interleukin 6 (IL-6) and tumour necrosis factor alpha (TNF-α), while suppressing anti-inflammatory adipokines like adiponectin [54]. The resulting chronic low-grade inflammation activates intracellular inflammatory pathways such as the nuclear factor-kappa B (NF-κB), further perpetuating a state of systemic inflammation [55]. This inflammatory environment contributes to oxidative stress and mitochondrial dysfunction, key mediators of insulin resistance [56,57]. Elevated levels of reactive oxygen species (ROS), which exceed the neutralizing capacity of cellular antioxidants, impair insulin signalling by activating stress-sensitive serine kinases such as JNK and IKKβ. These kinases phosphorylate insulin receptor substrate (IRS) proteins on serine residues, inhibiting downstream insulin signalling and promoting insulin resistance [58,59]. Moreover, the impaired secretion of adiponectin, an insulin-sensitizing hormone, further diminishes insulin sensitivity, increasing the risk for development of glucose intolerance and T2DM [60]. T2DM is preceded by prediabetes. The intricate relationship between obesity and prediabetes is critical for preventing the progression to overt diabetes; yet, prediabetes is an understudied condition, and therefore the exact relationship is less established.

### 3.3 Prediabetes: A precursor for type 2 diabetes mellitus

**Fig. 2:**
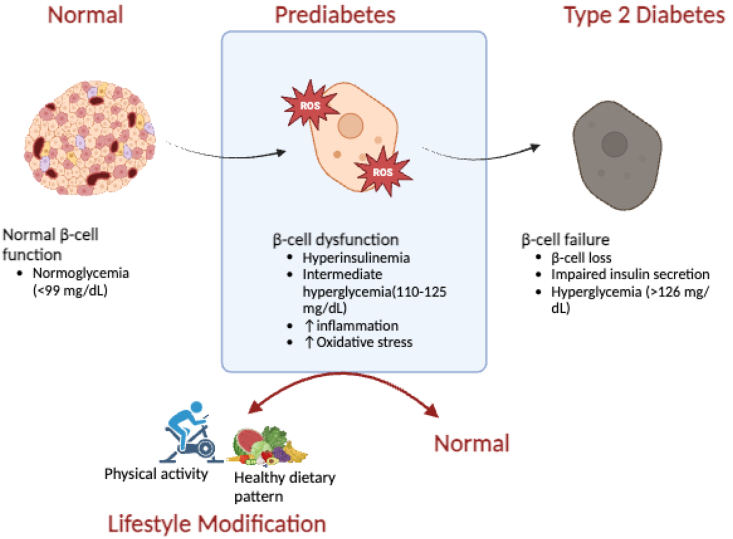
Prediabetes: pathophysiology, progression to T2DM and first-line lifestyle modification interventions for prevention; T2DM, Type 2 diabetes mellitus (created in BioRender).

Prediabetes is an asymptomatic metabolic condition in which an individual's blood glucose concentration is elevated above the normal range yet does not meet the threshold for an overt diabetes mellitus diagnosis [29]. Despite its clinical relevance, the term prediabetes remains controversial due to inconsistencies in its definition and perceived implications [61]. Critics argue that the prefix “pre-” may suggest a benign or insignificant condition, leading the general public and even healthcare providers to disregard and underestimate this intermediate high-risk state. Additional concerns relate to the financial implications of diagnosis, the psychological burden, and questions about the effectiveness and safety of early treatment interventions [[62], [63], [64]]. While these concerns are legitimate, early screening for prediabetes remains essential and cannot be negated, as its progression significantly increases the risk of developing more pronounced metabolic conditions, such as T2DM [29,65]. The prevalence of diabetes mellitus has been on a rapid upsurge over the last few decades, with estimates by the International Diabetes Federation (IDF) indicating that 589 million people globally were living with diabetes in 2024, the majority of whom were diagnosed with T2DM. [66,67]. This alarming trend highlights that prediabetes often goes undiagnosed and is only detected once individuals progress to overt diabetes thresholds. Targeting individuals with prediabetes, which affects nearly three times as many people with T2DM, can help change this trajectory and reduce the overall burden on the healthcare system [68]. The global prevalence of prediabetes is relatively unknown and often reported with divergent estimates, primarily due to differences in diagnostic criteria as well as inadequate disease surveillance [69]. Current diagnostic criteria vary: the WHO defines prediabetes by impaired fasting plasma glucose (IFG) of 110–125 mg/dL and impaired glucose tolerance (IGT) of 140–200 mg/dL based on a 2-h oral glucose tolerance test (OGTT) [70], whereas the American Diabetes Association (ADA) uses the same IGT threshold, a lower IFG threshold of 100–125 mg/dL and additionally includes glycated haemoglobin (HbA1c) between 5.7 and 6.4 % [71,72]. The lack of one globally accepted diagnostic criterion that integrates both WHO and ADA guidelines compromises global screening efforts. Furthermore, these inconsistencies may lead to missed diagnoses or overdiagnosis due to overlaps in the diagnostic criteria [73]. The progression rate from prediabetes to overt T2DM is around 5–10 % per year. This relatively slow progression represents a critical window of opportunity for early intervention to improve the associated modifiable lifestyle risk factors in order to promote restoration to normoglycemia and halt the development of several metabolic complications associated with the inception of T2DM (Fig. 2) [74]. This risk increases parallel to increases in global obesity, which remains the key modifiable contributor [75]. The pathogenesis of prediabetes is multifaceted, and poorly understood, however, the complex interplay between insulin resistance and pancreatic β-cell dysfunction are considered central mechanisms [76]. Insulin resistance, caused by obesity-related ectopic fat and chronic inflammation, leads to increased blood glucose levels. Sustained elevated blood glucose levels result in the inadequate secretion of insulin by pancreatic β-cells required to compensate for insulin resistance and overcome glucose demands of the body, ultimately resulting in impaired glucose regulation [[77], [78], [79]]. The introduction of effective ART has allowed HIV-infected individuals to live longer; consequently as this population ages, the prevalence of age-related non-communicable diseases such as obesity has also increased [28]. Evidence suggests that HIV-infected patients frequently exhibit a higher prevalence of insulin resistance compared to the general population, placing them at an increased risk for prediabetes and T2DM [80]. The prevalence of insulin resistance among HIV-infected patients varies and ranges between 20 % and 50 %, depending on the ART regimen and demographic factors [81]. Ultimately, prediabetes and T2DM are characterized by varying degrees of pancreatic dysfunction driven by overlapping deleterious molecular mechanisms which implicate the pancreas. Among these, obesity-related lipotoxicity, oxidative stress, and systemic inflammation play significant roles. These pathological changes not only disrupt glucose metabolism but also significantly alter pancreatic structure and overall function. Consequently, the pancreas, encompassing both its endocrine and exocrine functions, emerges as a critical organ involved early in the progression of metabolic disease.

## 4 Pancreatic function in glucose homeostasis: endocrine-exocrine interplay

**Fig. 3:**
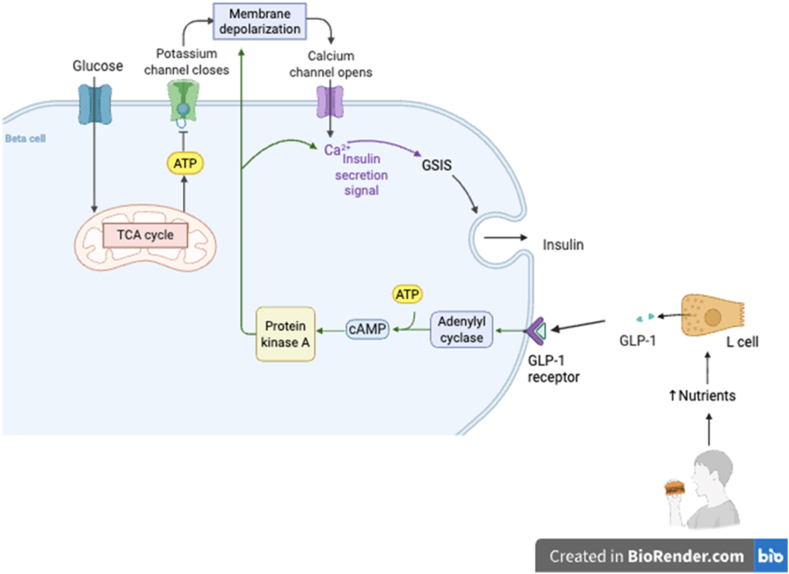
Synthesis and regulatory mechanisms of glucagon-like-peptide 1 (GLP-1) in glucose homeostasis. GLP-1 is synthesised by intestinal L-cells in response to nutrient stimuli and activates its receptor on pancreatic β-cells. This activation increases intracellular cyclic adenosine monophosphate (cAMP) levels and triggers pathways including protein Kinase A, potentiating glucose-stimulated insulin secretion (GSIS). ATP, adenosine triphosphate; TCA, tricarboxylic acid cycle*.*

Pancreatic health is essential due to the vital functions performed by the pancreas that tightly maintain glucose homeostasis and metabolic processes. The pancreas is a heterocrine gland, consisting of both exocrine and endocrine components, which together play a dual role in regulating digestion and blood glucose concentrations, respectively [82]. This dual functionality enables the efficient conversion of ingested nutrients, particularly carbohydrates, into energy that is subsequently absorbed into the bloodstream and utilized by other cells for key physiological functions throughout the body. Following nutrient digestion, blood glucose levels are regulated through the coordinated secretion of insulin by the pancreatic β-cells; this glucose-dependent insulin response is augmented by incretin hormones (Fig. 3) [83]. In the pancreatic β-cells, mitochondria are responsible for coupling glucose metabolism to insulin secretion through facilitating intermediary metabolic pathways that synthesize metabolites which support glucose-stimulated insulin secretion (GSIS) [84,85]. Glucose enters the pancreatic β-cells passively via the insulin-independent GLUT2 transporter and is phosphorylated by glucokinase to form glucose-6-phosphate. This metabolite is further processed through glycolysis, yielding pyruvate, which then shuttles into the mitochondria to fuel the tricarboxylic acid (TCA) cycle and the electron transport chain (ETC), ultimately generating adenosine triphosphate (ATP) [53,84]. Rising ATP levels inhibit the ATP-sensitive potassium (K_ATP_) channel, leading to membrane depolarization and the activation of voltage-gated calcium (Ca^2+^) channels. The resulting influx of intracellular Ca^2+^ triggers insulin exocytosis through promoting the fusion between the vesicle containing insulin and the pancreatic β-cell membrane, thereby facilitating glucose regulation [84]. However, chronic metabolic stress, particularly in the context of obesity and insulin resistance, can disrupt these finely tuned interdependent processes. Prolonged exposure to elevated circulating lipids and glucose, known as glucolipotoxicity, alters mitochondrial activity, increases oxidative stress through the excessive production of ROS and impairs ATP synthesis, ultimately accelerating pancreatic β-cell dysfunction [86,87]. Despite being distinct entities, the pancreas is an integrated organ, with the endocrine and exocrine components interacting and coexisting in harmony [88]. Consequently, any condition affecting either the exocrine or endocrine pancreas may ultimately affect the other due to their close anatomical and functional connections [89]. These glucolipotoxicity-induced disruptions may not only compromise endocrine pancreatic function but may also extend to involve the exocrine pancreas.

## 5 Metabolic-dysfunction associated steatotic fatty pancreatic diseases

The term metabolic dysfunction-associated steatotic fatty pancreatic disease (MASPD) is not currently an accepted term for describing the accumulation of fat in the pancreas without any history of alcohol consumption [90]. The current and widely recognised term for this condition is non-alcoholic fatty pancreatic disease (NAFPD) [91]. However, this review extrapolates MASPD from the term metabolic dysfunction-associated steatosis liver disease (MASLD), which is predominantly used to describe fatty liver disease unrelated to alcohol intake [92]. These conditions may occur in different organs and display different symptoms; however, they share a common feature of ectopic fat accumulation, also known as steatosis [39,93]. A significant number of pancreatic steatosis cases are associated with conditions of metabolic dysfunction including obesity and systemic insulin resistance [93]. However, whether insulin resistance precedes the pancreatic steatosis remains unclear. The current literature has limited profiling of MASPD progression from pancreatic steatosis to pancreatic cancer. This may be due to the lack of established diagnostic criteria and absence of standardised screening tests. Hence, the global prevalence of MASPD remains largely unknown [94,95]. Existing epidemiological data are limited and primarily derived from studies within Asian population, where prevalence estimates range between 16 % and 35 % [94]. Furthermore, this limited literature on MASPD may be attributed to the shortage of longitudinal studies that investigate the aetiology of pancreatic steatosis in the absence of obesity. This lack of longitudinal studies further impacts the literature that describes more risk factors for MASPD. Currently, obesity is regarded as the major risk factor associated with the development of MASPD [94]. Given this strong association, the global prevalence of MASPD is expected to increase in parallel with the rising global prevalence of obesity. Additional risk factors such as age, gender, and ethnicity have also been implicated, though their specific individual contributions to the development or progression of MASPD remain relatively unclear [91]. However, MASPD may represent an underrecognized yet clinically relevant manifestation of metabolic syndrome [96]. Hence, other metabolic risk factors of MASLD, such as impaired glucose tolerance, might need to be studied in relation to the onset of MASPD. The diagnostic methods used to detect MASPD/NAFPD include ultrasonography, computed tomography, magnetic resonance imaging, and magnetic resonance spectroscopy, which are used to evaluate pancreatic fat [91,97]. However, there is no consensus on a single optimal diagnostic method due to varying advantages, limitations, and cost implications associated with each technique [94]. Furthermore, not all fatty pancreas denote MASPD; therefore, differential diagnosis may be critical, particularly in ruling out alternative causes of pancreatic fat replacement, which may include alcohol-induced effects [94]. Although extensive literature on MASPD remains limited, its strong link to obesity highlights the importance for further research to elucidate its pathophysiology and clinical significance. The relationship between ART and MASPD is poorly understood and warrants investigation. Longitudinal studies examining the connection between ART and MASPD are scarce. Additionally, imaging studies quantifying pancreatic fat in PLHIV using histology or biopsy are limited. The majority of existing research on MASPD has been conducted in the general population or in relation to other conditions such as diabetes, with minimal focus on HIV-specific cohorts [[98], [99], [100], [101]]. Moreover, current literature tends to address pancreatic abnormalities in HIV patients, such as pancreatitis, rather than pancreatic fat accumulation [102]. Ultimately, the accumulation of fat in the pancreas and subsequent pancreatic β-cell dysfunction due to lipotoxicity directly impact the regulation of glucose metabolism through various mechanisms such as inflammation and oxidative stress. This inhibits glucose-stimulated insulin secretion (GSIS), promoting the development of postprandial hyperglycaemia and eventually T2DM [86].

## 6 Pancreatic incretin resistance

Postprandial hyperglycaemia, a metabolic disturbance frequently observed in obesity, contributes significantly to the development of T2DM [103,104]. Central to the regulation of postprandial glucose levels are incretin hormones, peptides secreted by specialized enteroendocrine cells in the gut: glucose-dependent insulinotropic polypeptide (GIP) from K-cells in the upper intestine, and glucagon-like peptide-1 (GLP-1) from L-cells in the lower intestine [105]. Together, these incretin hormones exert insulinotropic effects that are greatly involved in glucose metabolism and energy balance [106]. Incretin hormones are secreted within minutes of nutrient ingestion, predominantly carbohydrates, and stimulate pancreatic β-cells by binding to GIP and GLP-1 receptors on the cell membrane [107]. This interaction potentiates insulin secretion and suppresses glucagon release to meet the elevated glucose demands during the fed state [105]. The insulin secretory response following oral glucose intake accounts for approximately two-thirds than that following equivalent intravenously administered glucose, a phenomenon referred to as the incretin effect [103,105,106]. Additionally, incretin hormones are responsible for other physiological effects including β-cell proliferation and body weight regulation through central nervous system signalling pathways that delay gastric emptying and promote satiety, leading to low food intake and weight loss [103]. These beneficial effects have been suggested to be predominantly mediated by circulating GLP-1, while the majority of the insulinotropic effects involved in the incretin effect may be attributed to GIP activity [105]. The enzyme dipeptidyl peptidase 4 (DPP4) regulates incretin hormone activity by cleaving the N-terminal dipeptides and inactivating them, thus terminating postprandial insulin secretion [108,109]. Given their critical role, incretin-based therapies including GLP-1 receptor agonists and DPP4 inhibitors have become major treatment strategies for T2DM and obesity [83,110,111]. Paradoxically, despite excess glucose intake in obesity, the incretin system becomes impaired, contributing to metabolic complications. Lipotoxicity and insulin resistance induce pancreatic β-cell dysfunction, marked by reduced expression of GIP receptors on β-cell membrane [105]. This leads to incretin resistance, where the insulinotropic effect of GIP is reduced or inhibited despite its elevated secretion. Conversely, GLP-1 secretion decreases with increasing BMI [105]. Although GLP-1 receptors may be preserved, circulating GLP-1 levels alone may not be sufficient to maintain a normal incretin effect. Thus, the net result is an impaired incretin effect, leading to inadequate postprandial insulin secretion, sustained hyperglycaemia, and progressive pancreatic β-cell dysfunction, hallmarks of T2DM development [103,109]. The impact of ART on incretin hormone expression in PLHIV is not extensively documented. Limited studies indicate that glucose-intolerant HIV-infected individuals may exhibit enhanced GLP-1 responses to oral glucose stimuli compared to HIV-infected individuals with normal glucose tolerance [112], potentially representing a compensatory mechanism mediated by insulin resistance [113]. However, due to the life-long nature of HIV infection and its management, HIV-infected individuals commonly present with chronic inflammation, accompanied by injury and damage to enteroendocrine cells, which may eventually affect insulin secretion and insulin sensitivity [113].

## 7 ART regimens and general metabolic outcomes

Despite significant efforts to understand the HIV life cycle and the development of therapies that target key points of the viral life cycle to interfere with viral replication and increase viral suppression, HIV infection continues to constitute a public health challenge. According to the WHO, approximately 1.3 million people were newly infected with HIV in 2023. HIV infection impairs immunological function through viral replicative cycles that enable the HIV virion to invade host immune cells and evade host immune responses [114]. Over time, ART has evolved significantly. Early highly active antiretroviral therapy (HAART) regimens were associated with a high pill burden, which negatively impacted adherence and treatment outcomes [115]. In contrast, modern combination antiretroviral therapy (cART) regimens have substantially improved adherence and, hence, increased the life expectancy of PLHIV [116]. However, long-term cART use has been linked to metabolic complications, including insulin resistance, dysglycemia, hypertension, dyslipidaemia, and lipodystrophy [15]. According to the IDF, the concurrent occurrence of these metabolic derangements is collectively referred to as metabolic syndrome (MetS), which emphasises central obesity as a required component and a common phenotype in HIV-associated lipodystrophy [117,118]. Globally, the prevalence of MetS is increasing, especially among the aging HIV population on long-term ART [119]. A systematic review and meta-analysis reported a global pooled prevalence of MetS of 25.61 % among PLHIV receiving ART [120]. This condition is characterised by the coexistence of at least three metabolic disorders, including central obesity, hypertension, insulin resistance, and dyslipidaemia [121,122]. The pathogenesis underlying these metabolic complications is complex and multifactorial, involving the independent and synergistic effects of both the HIV infection itself and its chronic treatment using potent antiretroviral drugs, as well as interplay with other contributing risk factors such as genetic predisposition and age. HIV-infected individuals confer a higher risk of insulin resistance, which can be triggered directly by the virus through chronic immune activation and inflammation [28,123]. HIV-specific mechanisms, such as the inhibitory action of HIV-1 proteins on the peroxisome-proliferator-activated receptor-γ and heightened glucocorticoid sensitivity, further compound this risk. [[123], [124], [125]]. Chronic ART use adds to this burden, exacerbating HIV-related alterations, by inducing drug toxicity, which desensitises GLUT4 glucose transporters, impairs glucose uptake, and contributes to glucotoxicity and pancreatic β-cell dysfunction [123,126,127]. Together, these HIV- and ART-associated alterations in glucose and lipid metabolism promote low-grade inflammation and dysmetabolism, which fulfil the MetS criteria and consequently pose an increased risk for cardiovascular disease. Although HAART has been associated with dyslipidaemia, longitudinal studies investigating the chronic effects of cART treatment on the pathogenesis of MASPD remain limited(see Table 1).

**Table 1:** *A summary of the characteristics of the different antiretroviral drugs belonging to the two antiretroviral drug classes, NRTIs and NNRTIs and their metabolic effects*.

Antiretroviral drug class	Antiretroviral Drug/Regimen	Metabolic Effects
NRTI	Tenofovir disoproxil fumarate (TDF)	↑↑Renal biomarkers and bone mineral density [1] ↓LDL, HDL, and total cholesterol [2]
	Tenofovir alafenamide (TAF)	↓Renal biomarkers and bone mineral density [3] ↑↑LDL and HDL cholesterol, triglycerides [4] ↑↑Weight gain [5]
	Emtricitabine (FTC)	Generally metabolically neutral
NNRTI	Efavirenz (EFV)	↑↑Total cholesterol, triglycerides, HDL and LDL cholesterol [6] ↑↑Neuropsychiatric side effects [7,8] ↑Mitochondrial dysfunction [9] ↑Pro-inflammatory lipids [9] ↑Hepatic steatosis [9] ↑Hepatic transaminases [10]
	Rilpivirine (RPV)	↑ Total cholesterol, triglycerides, HDL and LDL cholesterol [6] ↑ Neuropsychiatric side effects [11] ↓ Pro-inflammatory lipids [12] ↑ Lipidomic markers [12] Hepatoprotective effects [13]: ↑ STAT-1 activation ↓ Hepatic stellate cell activation ↓ Liver fibrosis
	Doravirine (DOR)	↓Total cholesterol, LDL, triglycerides [14] ↑HDL cholesterol ↑ Neuropsychiatric side effects [8]

Key:*↑↑ = significant increase; ↑=minimal increase; ↓=decrease*.

*NRTI-nucleoside reverse transcriptase inhibitors; NNRTI-non-nucleoside reverse transcriptase inhibitors; LDL-low-density lipoproteins; HDL-high-density lipoproteins; STAT-1 -* signal transducer and activator of transcription-1.

## 8 HIV-ARV fixed-dose combinations

In the last few years, convenient once-daily cART regimens have become the standard of care for HIV treatment and prevention. These regimens are recommended as first-line therapy for both treatment-naïve and treatment-experienced individuals. In addition to their use in HIV management, once-daily fixed-dose combinations have also been approved by the FDA for use as PrEP in individuals at high risk of HIV infection. This includes cisgender men who have sex with men, transgender women who have sex with men, heterosexuals, and injection drug users [128,129]. The current Centers for Disease Control and Prevention (CDC) guidelines recommend PrEP for any sexually active individual at high risk of HIV infection. Modern ART is typically dispensed as single-tablet regimens that combine at least three antiretroviral drugs from two or more different antiretroviral classes. These drugs exhibit distinct molecular mechanisms to block HIV at various points of its life cycle, reducing the risk of drug resistance and efficiently achieving viral suppression. The currently recommended first-line single-tablet ART regimens usually consist of two nucleoside reverse transcriptase inhibitors (NRTIs) co-formulated with a third agent, which may be a non-nucleoside reverse transcriptase inhibitor (NNRTI), a boosted protease inhibitor (PI), or an integrase strand transfer inhibitor (INSTI). This review focuses specifically on NRTI/NNRTI-based regimens. Primarily, the mechanism of action for the NRTI and NNRTI classes is to inhibit HIV reverse transcription by targeting the HIV reverse transcriptase enzyme and thus preventing viral replication [130]. While modern antiretroviral drugs are generally well-tolerated and have improved safety profiles, long-term use has been associated with adverse events that may predispose HIV-infected individuals to various metabolic complications [131].

### 8.1 Nucleoside reverse transcriptase inhibitors

**Fig. 4:**
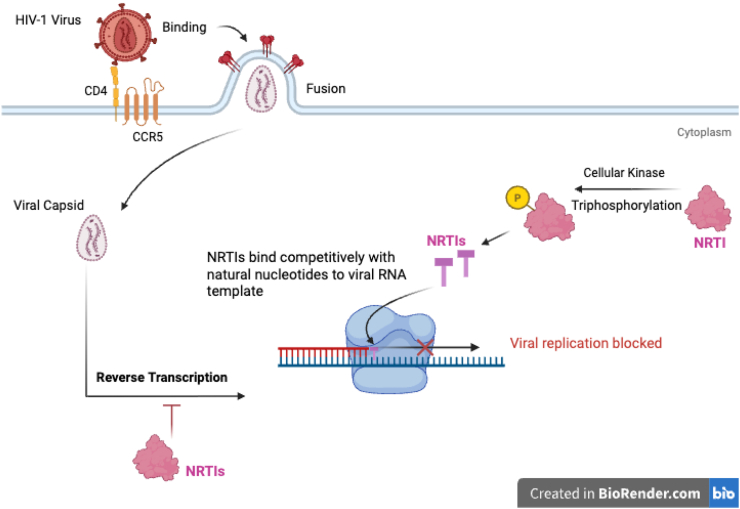
Mechanism of action of NRTIs: Cellular kinases phosphorylate NRTIs, converting them into their active triphosphate forms, which then compete with the natural deoxynucleoside triphosphates (dNTPs) to bind to the viral RNA template during reverse transcription. Their incorporation causes premature termination of the DNA chain, thereby blocking viral replication*.*

Nucleoside reverse transcriptase inhibitors (NRTIs) represent one of the earliest classes of antiretroviral drugs. Their development began with the discovery of zidovudine in the 1960s, initially investigated as an anticancer drug. Although unsuccessful in that role, zidovudine later emerged as an effective *anti*-HIV drug and was approved by the FDA in 1987 under the name azidothymidine [132]. NRTIs exert their antiviral activity through their pharmacologically active triphosphate metabolites. These metabolites competitively bind to and incorporate into the growing viral DNA chain, causing chain termination (Fig. 4). This occurs because NRTIs lack a 3′-hydroxyl group, which is essential for the continuation of viral DNA synthesis. As a result, viral replication is halted [132]. The NRTIs form the backbone of cART regimens due to their high efficacy profile. However, their use is associated with adverse effects indicative of mitochondrial toxicity, which may lead to myopathy, hepatic steatosis, lactic acidosis, or lipoatrophy [133]. The incidence of these metabolic complications is substantially lower with newer NRTI regimens [133,134]. Clinically FDA-approved NRTIs available for use in combination as part of single-tablet regimens to effectively suppress HIV replication include *lamivudine* (3 TC), *abacavir* (ABC), *tenofovir disoproxil fumarate* (TDF), *tenofovir alafenamide* (TAF), and *emtricitabine* (FTC). These single-tablet regimens may also be administered for the management of hepatitis B virus (HBV) infection; however, 3 TC-based regimens should be administered concomitantly with an HBV-active drug in HIV/HBV coinfection.

#### 8.1.1 Tenofovir

Tenofovir is an *anti*-HIV nucleotide analogue of adenosine 5′-monophosphate, first described and FDA-approved in 1985 [132]. As a monophosphate analogue, tenofovir requires sequential intracellular phosphorylation to its active form, tenofovir-diphosphate, which inhibits reverse transcriptase [135]. Due to tenofovir's low oral bioavailability and poor membrane permeability, attributable to its polar phosphonic acid group, two prodrug formulations tenofovir disoproxil fumarate (TDF) and tenofovir alafenamide (TAF) were developed to improve absorption and plasma drug levels [132,135]. These formulations are widely used in cART regimens for HIV treatment and prevention. Clinical studies have shown TDF- and TAF-based regimens to be non-inferior in efficacy and safety. However, these regimens present varying adverse effects [136].

##### 8.1.1.1 1Tenofovir disoproxil fumarate

Following oral administration, TDF is efficiently absorbed in the gastrointestinal tract and rapidly metabolised to tenofovir in plasma [135]. Tenofovir then penetrates target cells, such as T lymphocytes and macrophages, where it is phosphorylated to the active tenofovir-diphosphate form. This metabolite inhibits HIV reverse transcriptase, thereby blocking viral replication [135]. TDF-based regimens have been associated with adverse effects including liver toxicity, decreased bone mineral density, and the development or progression of renal impairment due to low intracellular and elevated plasma tenofovir concentrations, which circulate in the renal proximal tubules and lead to tubular dysfunction [1,132,135].

##### 8.1.1.2 2Tenofovir alafenamide

Tenofovir alafenamide (TAF) was developed as an improved prodrug of tenofovir and is considered superior to TDF despite non-inferior efficacy [137]. In contrast to TDF, the pharmacokinetics of TAF result in higher intracellular levels of tenofovir-diphosphate with significantly lower plasma concentrations of tenofovir, which significantly reduce renal and bone adverse events [135,137]. Despite its improved safety profile, TAF is associated with metabolic complications, including increases in total cholesterol, low-density lipoproteins (LDL), high-density lipoproteins (HDL), triglycerides, fasting glucose levels, as well as weight gain [4,48]. These findings indicate that chronic use of TAF-based regimens may increase the risk of dyslipidaemia, T2DM, and obesity.

#### 8.1.2 Emtricitabine

Emtricitabine (FTC), approved by the FDA in 2003, is a fluorinated cytidine analogue that is widely used in combination with other antiretroviral agents for HIV treatment and prevention as PrEP, combined with TDF in *Truvada* or TAF in *Descovy* [138,139]. Similar to most NRTIs, FTC is metabolised intracellularly to emtricitabine 5′-triphosphate, which inhibits HBV DNA polymerase and HIV reverse transcriptase, thereby blocking viral replication. In contrast to older NRTIs, the binding affinity of FTC for mitochondrial DNA polymerase-γ is low, resulting in minimal mitochondrial toxicity and a low incidence of lactic acidosis [132]. Hence, FTC has been reported as generally well tolerated and is considered lipid-neutral, with no significant metabolic complications reported.

### 8.2 Non-nucleoside reverse transcriptase inhibitors

**Fig. 5:**
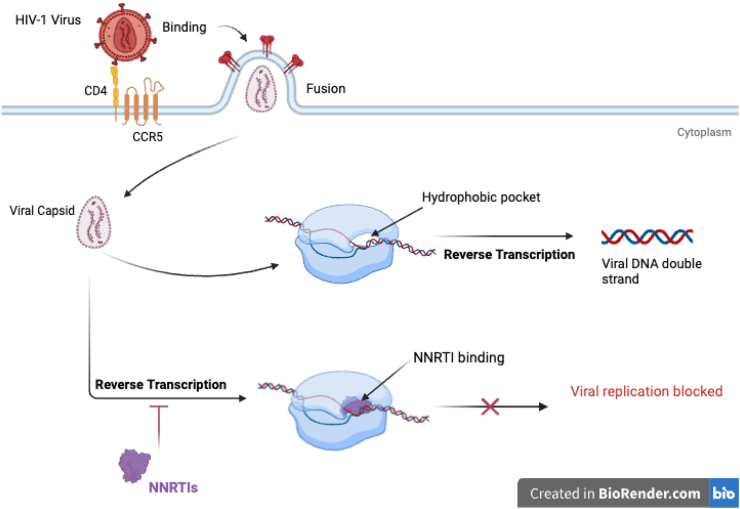
Mechanism of action of NNRTIs: By binding directly to HIV reverse transcriptase enzyme, NNRTIs allosterically block its active site, preventing the conversion of viral RNA into DNA and thereby inhibiting viral replication*.*

The Non-nucleoside reverse transcriptase inhibitors (NNRTIs) class of antiretroviral drugs, introduced in 1996, comprises of small hydrophobic chemically diverse drugs which directly inhibit the activity of the viral heterodimeric reverse transcriptase enzyme by complexing to its allosterically located hydrophobic pocket binding site (Fig. 5) [140]. This interaction induces a conformational change that alters and inhibits the catalytic activity of the active site, thereby preventing HIV replication [140]. Unlike NRTIs, this direct inhibition mechanism elicited by NNRTIs does not require the sequential intracellular phosphorylation of NNRTIs to convert to their active metabolites, as they already exist in their active forms [140]. Additionally, NNRTIs are highly specific and, therefore cannot be used in the management of other viral infections such as HBV. Clinically FDA-approved NNRTIs available for use in combination as part of single-tablet regimens to effectively suppress HIV replication include *efavirenz* (EFV), *nevirapine* (NVP), *rilpivirine* (RPV), and *doravirine* (DOR).

#### 8.2.1 Efavirenz

Efavirenz (EFV), the third FDA-approved first-generation NNRTI drug, was established in 1998 for the treatment of HIV [140]. Efavirenz is widely distributed in combination with other antiretroviral agents, particularly NRTIs such as TDF and FTC, in low and middle income countries [141]. Although EFV is generally well tolerated, it undergoes hepatic metabolism primarily through the cytochrome P450 isoenzyme 2B6 (CYP2B6) system, with minor pathways via the CYP3A4 and CYP2A6. This metabolic pathway is susceptible to drug-drug interactions, particularly with other antiretroviral agents or medications for comorbidities that inhibit these enzymes [142]. Moreover, inter-individual genetic variation in CYPP2B6 may significantly alter enzyme activity, resulting in higher plasma and intracellular EFV levels [143]. These elevated EFV levels are associated with an increased risk of toxicity, including neuropsychiatric side effects, hepatotoxicity, and lipid derangements [[143], [144], [145]]. Notably, the central nervous system (CNS) side effects linked to initiation or chronic use of EFV-based regimens have been reported to occur in over 40 % of HIV-infected patients [7]. HIV patients receiving EFV-based therapy demonstrate hepatotoxicity rates of approximately 8 % [146]. In contrast to some NRTIs, EFV has been linked to moderate mitochondrial dysfunction through mechanisms that directly impact mitochondrial function, potentially causing liver damage [9]. This dysfunction, specifically a depolarization of the mitochondrial membrane potential, can result in the reduction of ATP production, increased oxidative stress, inflammation, and ultimately apoptosis [9,147]. This bioenergetic stress in the liver can induce excessive fat accumulation, resulting in hepatic steatosis and consequently an increased risk for MASLD [9]. These mechanisms have been demonstrated in both *in vitro* and *in vivo* models. Although these models are valuable for studying mechanistic links, their findings do not fully represent the heterogeneity of the metabolic derangements in PLHIV due to the complexity and variability among patients. Therefore, large clinical studies are necessary to provide a more comprehensive understanding of the long-term effects of ART in PLHIV. Additionally, severe elevations in hepatic transaminases have been reported in up to 48 % of HIV-infected individuals on EFV-based regimens. These hepatic transaminase increases have been suggested to result from infrequent hypersensitive reactions following commencement of treatment or the gradual accumulation of EFV within hepatic tissue [10].

#### 8.2.2 Rilpivirine

Rilpivirine (RPV) is a second-generation NNRTI approved by the FDA in 2011 for use in combination with two NRTIs to treat HIV, primarily in HIV-infected individuals with pretreatment viral loads of less than 100 000 copies/mL. Despite these virologic limitations, RPV has been linked to substantially improved safety and metabolic profiles, with modest increases in lipid levels as well as lower incidence of dyslipidemia and CNS events [6,148,149]. Predominantly metabolised by the hepatic cytochrome P450 enzyme CYP3A4, RPV exhibits low systemic free plasma concentrations and minimal inhibitory effects on other drug-metabolising enzymes and transporters, resulting in a reduced potential for clinically significant drug-drug interactions [150]. Notably, clinical trials comparing 25 mg RPV and 600 mg EFV regimens demonstrated that RPV was superior to EFV when administered to those with lower baseline viral loads (≤100 000 copies/mL) and equally effective as EFV in those with higher baseline viral loads (100 000 < viral load <500 000 copies/mL) [151,152]. A mass spectrometry-based lipidomic profiling study has also found that switching from EFV-based therapy to RPV-based therapy reduces pro-inflammatory lipids and enhances lipidomic markers [12]. Moreover, switching to RPV-based regimens could potentially provide hepatoprotective effects through increasing signal transducer and activator of transcription (STAT) 1-mediated inactivation and apoptosis of hepatic stellate cells, thereby reducing hepatic fibrosis markers, improving liver function, and modulating MASLD in HIV-infected individuals [13].

#### 8.2.3 Doravirine

Doravirine is a novel second-generation NNRTI approved by the FDA in 2018 for treatment of HIV in combination with two NRTIs, TDF and lamivudine (3 TC). Similar to RPV, its metabolism is primarily mediated by the cytochrome P450 enzyme CYP3A4, resulting in low potential for drug-drug interactions [153,154]. The DRIVE-AHEAD clinical trial compared the efficacy and safety of DOR-based regimens to EFV-based regimens in treatment-naïve adults [14]. The results showed that DOR-based regimens were as virologically effective as EFV regimens, with lower weight gain, fewer CNS events and significantly reduced and sustained lipid improvements [14,155]. Given its well-tolerated nature, safety and efficacy profiles, lack of evident therapeutic limitations, and stability in the presence of common NNRTI resistance mutations, DOR-based therapy is considered superior to older drugs in the same class [156].

## 9 Implications for clinical practice: screening and monitoring prediabetes in HIV patients

Disease prevention is one of the four basic goals of medicine and healthcare, aligning closely with Sustainable Development Goal three (SDG-3), which emphasises good health and well-being. Central to this goal is the timely identification of at-risk populations and the implementation of preventative strategies to delay or prevent disease onset. PLHIV are at higher risk of prediabetes, a precursor to T2DM, due to the direct metabolic effects of HIV infection, which contribute to chronic inflammation [157], and the long-term use of ART, which has been evidenced to potentiate inflammation, leading to insulin resistance. Given this heightened risk, routine screening for prediabetes and the annual monitoring of glycemic levels in HIV-infected individuals are therefore imperative. Screening methods include fasting blood glucose (FBG), 2-h plasma glucose via a 75 g OGTT, and glycated haemoglobin (HbA1c) [158]. While HbA1c is widely used to assess glycemic status, its reliability in the HIV population may be impeded [159,160]. Measurement of HbA1c reflects average blood glucose levels over the preceding 2–3 months. Individual-specific risk factors such as age, race, and ethnicity, as well as other clinical conditions including HIV-related chronic inflammation, haemodialysis-related hemolysis, ART, and ART-induced anaemia, may shorten the average lifespan of erythrocytes, limiting the time available for haemoglobin glycation and resulting in falsely low HbA1c values that underestimate the true glycemic status and the prevalence of prediabetes in this population [158,161]. Recognising these HbA1c limitations, both the European AIDS Clinical Society (EACS) and the ADA updated their guidance in 2023 and 2024, respectively, recommending that when inconsistencies are suspected between HbA1c and glucose-based assessments, FBG and OGTT should be prioritised or used in combination to improve diagnostic accuracy. Moreover, studies have also shown that combining FBG and HbA1c improves detection rates of prediabetes in HIV-infected individuals to approximately 75 % [162]. Taken together, a multi-test approach combining HbA1c performed by a National Glycohemoglobin Standardisation Program (NGSP)-certified method and standardised to the Diabetes Control and Complications Trial (DCCT) assay, with FBG and OGTT, offers a more accurate assessment of dysglycemia in PLHIV, reducing underdiagnosis and enabling timely intervention in this population [158].

### 9.1 Considerations in antiretroviral drug selection

The metabolic impact of ART varies widely among individuals due to differences in virologic, immunologic, behavioural, and pharmacologic factors [163]. This variability underscores the necessity for individualize treatment plans [164]. When selecting HIV therapies, clinicians must consider patient-specific factors such as age, genetic predispositions, medical history, potential drug interactions, and therapeutic goals. Given the side effect profiles of the approved and widely used NRTIs and NNRTIs, which are recommended as first-line therapy, identifying risk factors that may be exacerbated by the chronic use of specific drugs should be a priority. This highlights the need for personalized therapy tailored to individual patient risks. The use of TDF-based regimens should be avoided in patients with pre-existing renal dysfunction, as these drugs can accelerate renal impairment and potentially lead to chronic kidney disease. In contrast, TAF-based regimens are associated with a more favourable renal safety profile; however, the use of these regimens necessitates baseline screening of body weight, BMI, and lipid profiles. This is because TAF use may contribute to elevated lipid levels and increased body weight, which can predispose individuals to dyslipidaemia and obesity, requiring careful metabolic monitoring. Caution should be taken when initiating NNRTI-based regimens, especially those that include EFV, in patients with pre-existing neuropsychiatric disorders. A thorough neuropsychiatric assessment should be conducted prior to treatment initiation, and in severe instances, EFV should be avoided to prevent exacerbation of neuropsychiatric symptoms. Moreover, prior to EFV treatment initiation, liver function tests and lipid profiles are recommended. RPV- and DOR-based regimens are generally well tolerated. However, RPV should be avoided in patients with high baseline viral loads, as its efficacy may be reduced in this population. Furthermore, RPV requires administration with food to ensure optimal absorption and therapeutic levels. PrEP is primarily used by HIV-negative individuals engaged in high-risk sexual behaviours. Therefore, before administration of PrEP, HIV testing must be performed to confirm that the individual is not already infected. This screening is crucial to avoid drug resistance, which could limit future antiretroviral treatment options [165].

### 9.2 Strategies for minimising metabolic complications in population living with HIV

Given the well-documented increased prevalence of metabolic complications associated with HIV and its treatment, prioritising prevention and management strategies is essential. In conjunction with routine blood glucose level monitoring, comprehensive metabolic assessments should be conducted regularly to prevent serious microvascular and macrovascular complications such as diabetic retinopathy, kidney disease, peripheral neuropathy, foot ulcers, and cardiovascular disease [166,167]. At the time of HIV diagnosis, rigorous screening and prevention strategies must be implemented. Preventative measures include counselling by trained healthcare providers to support emotional well-being and promote drug adherence, which are essential for optimal health outcomes. Following counselling, medical nutrition therapy is necessary to encourage sustainable lifestyle changes that facilitate weight loss and potentially induce diabetes remission. Baseline screening of glucose and lipid profiles before initiating HIV treatment is crucial to establish metabolic health status, with at least annual follow-up screenings recommended to monitor changes and further guide management. Ongoing care should include yearly ophthalmologic examinations to detect early retinopathy, maintenance of optimal blood pressure and lipid levels, urine microalbumin testing every twelve months, and foot examinations every six to twelve months to prevent complications [166,167]. In cases where metabolic complications progress, antidiabetic medicines such as metformin should be prescribed cautiously, with careful monitoring for potential adverse drug interactions with ART [167]. Medication therapy should be tailored to individuals’ evolving clinical status, with opportunities for switching regimens to optimise health outcomes.

## 10 Conclusion and perspectives

**Fig. 6:**
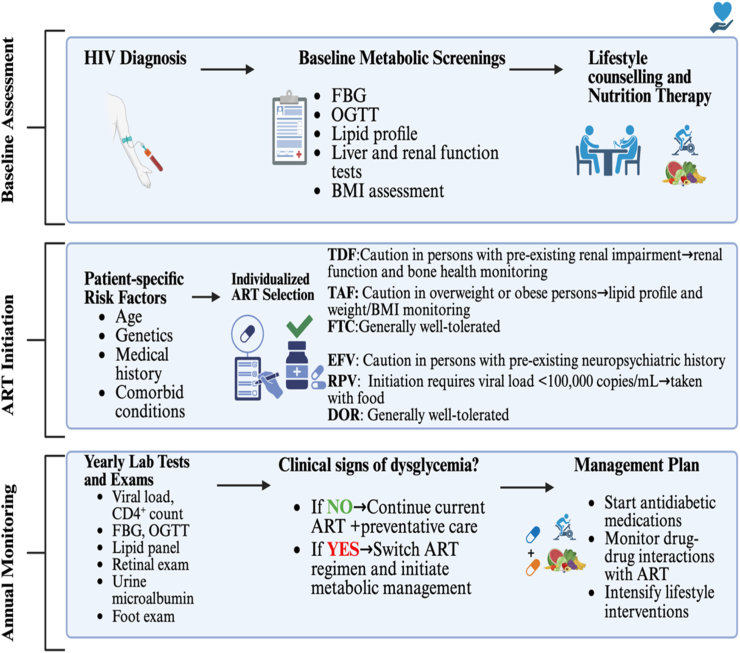
Schematic overview of HIV management: From baseline assessment to ART initiation decision-making and long-term monitoring (created in BioRender).

The intersecting burden of HIV and obesity-linked metabolic disease is a growing public health concern. PLHIV are increasingly at risk of developing prediabetes and T2DM, driven by a combination of metabolic effects, including ageing, chronic HIV infection, and modifiable lifestyle risk factors. This burden is further compounded by ART-specific factors, including drug-related toxicities related to the unavoidable prolonged treatment duration. The evolution of ART has contributed in the reduction of side effects including metabolic. Classes of ART including NRTIs continue to serve as the backbone of HIV-ART regimens globally. The recent NRTIs such as TDF has been shown to generally exhibit a favourable metabolic profile, however, posing a risk of chronic kidney diseases. Conversely, TAF, another tenofovir-releasing prodrug, offers improved renal and bone safety but is associated with significant metabolic alterations, including weight gain and lipid disturbances, which may predispose HIV-infected individuals to an overweight status and, with prolonged use, progress to obesity and related lipid disorders. Hence, there is a need to investigate the interaction of the NRTIs with specific organs in the body to develop a potent drug that is less harmful. The NNRTIs-based HIV-ART regimens continue to be prescribed, particularly in resource-limited settings or in specific clinical scenarios where patient risk profiles warrant their prescription. NNRTIs such as Efavirenz are now less frequently employed as initial ART due to their association with adverse effects, including neuropsychiatric and low resistance barrier. However, more NNRTIS are being developed, such as Doravirine and rilpivirine, which remain viable alternative options in the HIV management. While the efficacy of cART is undisputed, certain metabolic challenges persist, requiring ongoing vigilance for adverse effects to optimise patient care. For patients still on NRTI/NNRTI-based regimens, an integrated approach to HIV management that prioritises early identification and prevention of metabolic derangements is essential. This approach should include targeted lifestyle modifications, comprehensive metabolic screening and monitoring, and individualised ART selection to optimise and improve long-term health outcomes for PLHIV (Fig. 6).

## 11 Knowledge gaps and future directions

Despite the pancreas's critical role in glucose homeostasis and T2DM pathogenesis, its response to long-term HIV treatment remains underexplored, particularly in the context of intersecting lifestyle-driven metabolic alterations. Chronic use of NRTI/NNRTI-based regimens, combined with lifestyle risk factors and non-modifiable risk factors such as age, may promote fat deposition in the pancreas. This ectopic pancreatic fat accumulation can impair both endocrine and exocrine functions, disrupt hormonal signalling pathways such as insulin resistance and incretin hormone activity, and accelerate the progression from prediabetes to overt diabetes. This review highlights prediabetes as a significant yet often neglected component in the interplay between HIV, ART, and T2DM. Prediabetes is frequently undiagnosed at ART initiation, meaning many patients start treatment with unaddressed metabolic risks. Research on how chronic NRTI/NNRTI regimens affect pancreatic function and the transition from prediabetes to diabetes is limited. Future studies should prioritise animal models and clinical investigations focusing on the specific impact of these regimens on pancreatic function, tissue morphology, and gut incretin regulation. Moreover, not only patients on NRTI/NNRTI-based regimens but also those receiving regimens widely recommended as first-line therapies, which are used by large populations, require rigorous documentation of their metabolic safety profiles. Since prediabetes is reversible, understanding these mechanisms is crucial for developing targeted interventions, improving screening and treatment strategies, and ultimately enhancing clinical outcomes for PLHIV. Such research will also contribute to achieving UNAIDS 95-95-95 targets and addressing the growing dual burden of HIV and noncommunicable diseases in this population.

## Ethics approval and consent to participate

The data that will be analysed will be the data that is published and there will be no data collection from subjects. The authors declare that there will be no informed consent required to be signed and therefore no ethics approval required for the narrative review.

## Consent for publication

Not applicable.

## Availability of supporting data

No extra data available besides the attached additional file since it is a narrative review.

## CRediT authorship contribution statement

**Amogelang Sedibe:** Conceptualization, Investigation, Methodology, Project administration, Software, Validation, Visualization, Writing – original draft, Writing – review & editing. **Ntethelelo Sibiya:** Conceptualization, Methodology, Supervision, Validation, Visualization, Writing – review & editing. **Trevor Nyakudya:** Conceptualization, Methodology, Supervision, Validation, Writing – review & editing. **Mlindeli Gamede:** Conceptualization, Funding acquisition, Investigation, Methodology, Project administration, Supervision, Validation, Visualization, Writing – review & editing.

## Declaration of competing interest

The authors declare that they have no known competing financial interests or personal relationships that could have appeared to influence the work reported in this paper.

## Figures and Tables

**Table:** 

ADA	American Diabetes Association
ART	antiretroviral therapy
ARV	antiretroviral
ATP	adenosine triphosphate
BMI	body mass index
CART	combination antiretroviral therapy
CNS	central nervous system
DOR	doravirine
DPP4	dipeptidyl peptidase 4
EFV	efavirenz
FDA	U.S. Food and Drug Administration
FTC	emtricitabine
GIP	glucose-dependent insulinotropic peptide
GLP-1	glucagon-like Peptide-1
GLUT-4	glucose transporter-4
GSIS	glucose-stimulated insulin secretion
HAART	highly active antiretroviral therapy
HbA1c	haemoglobin A1c
HBV	hepatitis B Virus
HDL	high-density lipoprotein cholesterol
HIV	human immunodeficiency virus
IDF	International Diabetes Federation
IFG	impaired fasting glucose
IGT	impaired glucose tolerance
LDL	low-density lipoproteins
MASLD	metabolic dysfunction associated steatotic liver disease
MetS	metabolic syndrome
NAFLD	non-alcoholic fatty liver disease
MASPD	metabolic dysfunction associated steatotic fatty pancreas disease
NNRTI	non-nucleoside reverse transcriptase inhibitors
NRTI	nucleoside reverse transcriptase inhibitors
OGTT	oral glucose tolerance test
PLHIV	people living with HIV
PrEP	pre-exposure prophylaxis
ROS	reactive oxygen species
RPV	rilpivirine
TAF	tenofovir alafenamide
3 TC	lamivudine
TDF	tenofovir disoproxil fumarate
T2DM	type 2 diabetes mellitus
UNAIDS	Joint United Nations Programme on HIV/AIDS
WHO	World Health Organisation

## Data Availability

No data was used for the research described in the article.
